# Chimpanzee vowel-like sounds and voice quality suggest formant space expansion through the hominoid lineage

**DOI:** 10.1098/rstb.2020.0455

**Published:** 2022-01-03

**Authors:** Sven Grawunder, Natalie Uomini, Liran Samuni, Tatiana Bortolato, Cédric Girard-Buttoz, Roman M. Wittig, Catherine Crockford

**Affiliations:** ^1^ Department of Human Behavior, Ecology and Culture, Max Planck Institute for Evolutionary Anthropology, Deutscher Platz 6, 04103 Leipzig, Germany; ^2^ Department of Linguistic and Cultural Evolution, Max Planck Institute for Evolutionary Anthropology, Deutscher Platz 6, 04103 Leipzig, Germany; ^3^ Department of Empirical Linguistics, Goethe University, Frankfurt am Main, Germany; ^4^ The Ape Social Mind Lab, Institut des Sciences Cognitives, CNRS, 67 Boulevard Pinel, 69675 Bron, Lyon, France; ^5^ Department of Human Evolutionary Biology, Harvard University, Cambridge, MA, USA; ^6^ Tai Chimpanzee Project, Centre Suisse de Recherches Scientifiques, 01 BP 1303, Ivory Coast

**Keywords:** evolution of language, formants, primate, speech, chimpanzees, hominoid

## Abstract

The origins of human speech are obscure; it is still unclear what aspects are unique to our species or shared with our evolutionary cousins, in part due to a lack of a common framework for comparison. We asked what chimpanzee and human vocal production acoustics have in common. We examined visible supra-laryngeal articulators of four major chimpanzee vocalizations (hoos, grunts, barks, screams) and their associated acoustic structures, using techniques from human phonetic and animal communication analysis. Data were collected from wild adult chimpanzees, Taï National Park, Ivory Coast. Both discriminant and principal component classification procedures revealed classification of call types. Discriminating acoustic features include voice quality and formant structure, mirroring phonetic features in human speech. Chimpanzee lip and jaw articulation variables also offered similar discrimination of call types. Formant maps distinguished call types with different vowel-like sounds. Comparing our results with published primate data, humans show less F1–F2 correlation and further expansion of the vowel space, particularly for [i] sounds. Unlike recent studies suggesting monkeys achieve human vowel space, we conclude from our results that supra-laryngeal articulatory capacities show moderate evolutionary change, with vowel space expansion continuing through hominoid evolution. Studies on more primate species will be required to substantiate this.

This article is part of the theme issue ‘Voice modulation: from origin and mechanism to social impact (Part II)’.

## 1. Introduction

The origins of human speech are obscure, and the order of emergence of components required for speech to evolve is much debated [1–5]. Here, we address the emergence of vowel sounds universal to human speech production. Given that speech and language do not fossilize, comparative research with other species can provide fruitful insights, and of particular relevance, pinpointing areas of consistency and divergence across the vocal repertoires of our closest living relatives, the non-human primates. Hampering comparative research is the continued lack of a common methodology for assessing human and non-human vocal production. Recent studies suggest that using human phonetic concepts that characterize vowel sounds, including formant analyses, can also be informative in describing vocal modulation in non-human primates [4,6,7]. Here, we combine human phonetic and animal acoustic approaches including lip, tongue and jaw articulation movements and formant assessment, where formants are the broad spectral peaks that result from acoustic resonances in the vocal tract [8,9]. These features together shape vowel sounds in humans. As such, we examine vocal modulation across the vocal repertoire in one of our closest living relatives, the chimpanzee (*Pan troglodytes verus*).

It was previously considered that the limited descent of the non-human primate larynx prevented variable production of vowel-like sounds [10]. Boë *et al*. [4,7], however, demonstrated that Old World monkeys, specifically baboons, naturally produce vocalizations with contrasting formant patterns. Models demonstrate that the formant patterns in non-human primates operate similarly to those of humans in terms of sounds produced. In source-filter theory of acoustic phonetics [11], all cavities above the larynx and the glottis (source) are considered as supra-laryngeal filters, influencing the spectrum of the source. Hence, this theory developed for the human vocal tract can be applied also to non-human primates (see [4] for a review). Formant extremes are shown to form a triangle in a two-dimensional acoustic space delineated by the first (F1) and second (F2) formants, with vowels [i, u, a] at the three extremes [12]. Most human languages use these extreme vowels in speech, presumably to gain maximum vowel contrast (H&H theory [13]).

Early non-human primate work applying the source-filter model [11,14] to Old World monkeys examined vocalizations in Diana monkeys [15], and chacma baboons [16]. Even though studies only addressed a limited part of the vocal repertoire for each species, alarm calls and grunts, respectively, they demonstrated modulation of the filter, namely resonance modulation resulting in formant configuration. Subsequent studies have shown similar results, particularly those that examine a broader range of vocalizations within each species' repertoire [4,6,7]. These studies demonstrate that monkeys attain vocalization variation through articulatory configurations, and refute the idea that the monkey vocal tract is a uniform tube with limited capacity to change formant patterns. Whilst humans show greater release than monkeys from the F1 to F2 correlation observed in a uniform tube, monkeys nonetheless demonstrate some limited relaxation of this constraint [7]. To demonstrate this, Boë *et al*. [4] plotted F1 and F2 for different non-human primate species, where available in the literature, onto the human formant space, correcting for vocal tract length. They showed that two monkey species each reach one vowel extreme in the human formant triangle space, but none of the non-human primate species examined reaches two or three of the vowel extremes. It should be noted that few studies examine F1 and F2 across the entire vocal repertoire of a species, with Boë *et al*. [7] being an exception. Hence, it is not clear if monkeys do not reach more than one vowel extreme or if this rather reflects limited research effort, likely due to difficulties in measuring formants, particularly in noisy environments where animal recordings are typically made.

Even though monkeys may show more limited formant use than humans, the capacity of monkeys to modify their formant patterns using articulations is nonetheless evident [6,7]. To understand what primates might gain from using vowel-like calls in their vocal repertoires, such as whether an expanded vowel space leads to a larger vocal repertoire, examination of the whole vocal repertoire per species is an advantage, but to date, few such studies exist [4].

In addition to formants, we are interested in the contribution of voice quality changes in distinguishing call types. The source modulation, i.e. the modulation and subsequent variation of the voice source (larynx with glottis) in the source-filter model, is acoustically described by parameters of periodicity or general trends of the frequency spectrum. These variations result in voice qualities which auditorily express the roughness, clarity, transparency or timbre of a voice [17]. Terms like ‘grunt’ versus ‘scream’ reflect some of these overall characteristics.

With respect to formant use, a highly relevant question is whether there has been selection through evolution for an expanded vowel space. To assess this idea requires including not only formant assessment across the vocal repertoire of monkey species, but also of species that are phylogenetically closer to humans, the great apes. To date, few studies have assessed great ape formant usage. Those that have are usually in relation to one or two call types within the vocal repertoire. These studies demonstrate formant structure (gorilla: double grunts [2]), or formant shifts, indicating that articulatory changes modulate both voiced and voiceless call production (chimpanzee: rough grunts given to foods of different preferences [18]; orangutans: voiceless clicks and voiced faux-speech [19]; grumph versus ‘wookie’ calls [20]). These studies suggest that articulatory movements, such as lip and jaw movements, likely contribute to call modulation in great apes, although the extent to which such movements assist in the classification of different call types across a species' repertoire has rarely been assessed.

One difficulty in assessing formants and other spectral features of vocalizations in great ape species is that great apes usually live in highly noisy environments, such as tropical forest, such that reliably extracting spectral features is challenging, especially using automatic classification approaches for low amplitude calls such as hoos and grunts [21]. Captive environments are also far from ideal for sound recording. In zoos and sanctuaries, when in out-door enclosures, recording distances are often greater than when following habituated wild animals, and for in-door settings, spectral features are often obliterated due to extensive echo or from human-imposed noise.

With these issues in mind, here, using a broad repertoire approach, we assess spectral features in two ways across chimpanzee vocalizations coded from audio files that have been extracted from video. First, we classify call types across the chimpanzee vocal repertoire adding classical temporal and frequency acoustic measures into both discriminant function analysis and principal components classification approaches. Second, we assess the impact of lip and jaw movement on call classification directly, using an ordinal scale of lip and jaw movements coded visually from video. In addition, we superimpose chimpanzee call types and their formant measures onto the human vowel space, as well as onto the formant space of other primates (after [4,6]). We use video and audio data from two communities of wild chimpanzees in the Taï National Park, Ivory Coast. We assess the contribution of acoustic features often explanatory in speech sound and animal call classification to classify the main four call types in the chimpanzee vocal repertoire (hoos, grunts, screams and barks), each of which is used widely across the chimpanzee vocal repertoire.

## 2. Methods

### (a) Study site and subjects

Videos were recorded ad libitum by L.S., C.C., R.M.W. and other members of the Taï Chimpanzee Project [22] from two habituated communities of wild chimpanzees in the Taï Forest, Ivory Coast (5°45′ N, 7°07′ W): East and South Group, between October 2013 and May 2016. From the original library of video recordings, we selected videos of identifiable chimpanzees vocalizing where the face, and specifically the mouth, of the signaller was visible during at least one vocalization. This produced a dataset of vocalizations from 28 adult and subadult chimpanzees (greater than 10 yr old) from two neighbouring chimpanzee communities: East Group—eight females and four males, South Group—nine females and seven males (electronic supplementary material, table S1). Video recordings were made with a HD Panasonic camera at 25 fps, 720 px image width.

### (b) Video analysis: assigning articulatory scores

In order to examine the visual articulators used during vocalizing, N.U. annotated all videos in ELAN 4.9 [23] a freeware (https://tla.mpi.nl/tools/tla-tools/elan/) which allows segmentation of visual and auditory signals to millisecond precision. We annotated the vocalizations of each individual into call bouts (labelled following four broad call types defined below), then further into breath units (BU), which are the units of analysis in this paper, defined below. An example screenshot of annotations is shown in electronic supplementary material, figure S1.

Chimpanzee vocalizations almost exclusively consist of a single vocalization per exhalation or inhalation (here, BU). Vocalizations, regardless of call type, can be produced as single BU or as a series of BU, either as sequential exhaled vocalizations or of alternating exhaled and inhaled (panted) vocalizations. Thus, barks, screams, grunts and hoos can all be emitted as single units or combined adjacent to panted units. Whether panted or unpanted, calls can likewise be classified as grunts or hoos and so on, thus for this study, we treated panted and unpanted versions of the same call type similarly. Call bouts can consist of repetitions of the same vocalization type (e.g. hoos or screams) or of combinations of different vocalization types (e.g. hoos + screams) [24]. A series of vocalizations with less than one-second pause are here considered to be part of the same *call bout* [24]. We measured the inter-call interval between combined calls for a random set of 314 bouts across the vocal repertoire. We found that the different calls are produced in rapid succession (0.23 ± 0.04 s, mean ± s.e.) within a bout. However, single grunts (e.g. emitted at food), are produced with wider intervals. In order not to artificially increase the number of bouts, we therefore used a one-second rule, as other studies have done [25]. In order to limit pseudoreplication, we randomly selected a maximum of two non-adjacent BU of the same call type within a call bout for each analysis, and medianized values of both BU in each bout.

**Figure 1.**
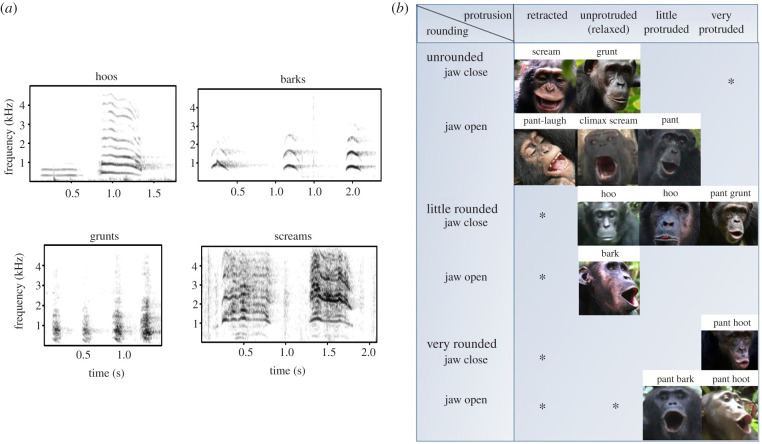
(*a*) Spectrograms of the four major chimpanzee vocalization types included. (*b*) Articulatory parameters visualizing the categorical coding scheme, with visual examples for each cell (lip protrusion and lip rounding: all categories represented; jaw position: 2 of 4 categories shown (fully closed (nasal emission), close (limited opening), mid, open (wide open), shown in electronic supplementary material, figure S3; see electronic supplementary material, table S2 for category definitions). Asterisk, not expected to occur/be feasible in the chimpanzee repertoire. Empty squares are expected to occur but were not represented in our sample. Photo credits: Liran Samuni, Cat Hobaiter.

For articulatory parameters, we devised a simple notation system to classify jaw and lip positions on a 4-point scale ranging from closed to wide open, rounded to unrounded, or retracted to protruded, respectively. We drew on principles applied to human vowels, inspired by the descriptive system of the International Phonetic Alphabet [26] (figure 1). The human system considers jaw opening and lip rounding, and we additionally distinguished lip protrusion, because we noted that chimpanzees can modify rounding independently from protrusion. We did not include tongue parameters because we could rarely see the chimpanzee tongue during vocalizing. The electronic supplementary material details our notation system with definitions. Inter-rater reliability scores between three coders (N.U., S.G., C.C.) for a subsample of 301 out of 1507 ratings annotated (three parameters on 529 articulatorily annotated BU) showed a good to high inter-rater reliability using the (two-way) average interclass correlation (ICC = 0.839, *F*_300,600_ = 6.21, *p* = 3.6 × 10^−80^, 95CI: 0.805 < ICC < 0.868) in irr R-package [27], indicating that the notation system is user-friendly.

### (c) Audio analysis: assigning call types

Sound tracks from the videos were extracted using ELAN, and ELAN annotations were loaded into PRAAT [28]. We then classified by ear each breath unit as a particular *call type*. Although the chimpanzee vocal repertoire is a graded system [21], such that most call types grade into other call types, call types can largely be differentiated by ear after a training period due to their distinctive auditory and acoustic features (table 1; see electronic supplementary material for sound files of each call type, electronic supplementary material, figure S2 for gradations of these call types).

**Table 1.** Number of breath units (BU) per call type for acoustic and articulatory data.

call type	BUs with acoustic measures only including two non-adjacent BUs per call type per call bout (*N* = 427)				BU with acoustic measures (*N* = 816)				BU with articulatory measures (*N* = 471)			
	hoo	grunt	bark	scream	hoo	grunt	bark	scream	hoo	grunt	bark	scream
number of BU	140	113	121	31	344	230	169	73	282	67	67	55
number of chimpanzees (>10 yr old)	18	20	24	12	18	20	24	12	7	10	10	7

Most calls were assigned to one of four broad call types that encapsulate most of the chimpanzee repertoire (hoos, grunts, barks and screams, [24,29,30]). For this analysis, less-commonly emitted whimpers, roars and pants were excluded because they were not well represented in this sample. Inhaled vocalizations were also omitted (specifically panted vocalizations between exhaled vocalizations). After these selection filters, the final dataset (corpus) consisted of 127 video clips, yielding 838 data points for 27 individuals (table 1). One hundred calls were subjected to inter-rater reliability with three blind coders to classify call types by ear (C.C., L.S., T.B.). After a training period, Kappa score reached a 94.6% of agreement on the call classification [31].

### (d) Acoustic analysis

We used two approaches to characterize the acoustic properties of the call types. First, we analysed the BU with successfully annotated articulatory measures. Second, after we extracted audio .wav files from video (both sampling rate/depth = 48 kHz/16bit), we included BU with good audio quality even when articulatory measures could not be coded, that is, when the face of the vocalizer was turned away from the video camera. We considered only calls of high quality, such that the lowest frequency band was visible and not obscured due to high background noise or overlap from other chimpanzee vocalizations. While this is a normal and necessary step for field recordings, here we had an additional constraint that video likely produces lower acoustic recording quality than the directional short or long gun microphones typically used in animal acoustic analyses. After this step, the quality of spectrograms was reasonably high and not notably different from audio-recordings from the same forest.

For the characterization of acoustic properties of call types, we chose the acoustic parameters listed below based on typical voice parameters used in human vocalization analysis [32]. These parameters best characterize the acoustic dimensions of voice in terms of sound energy structure and distribution and are potentially robust enough to serve an analysis of recordings taken from video. These recordings were then subjected to acoustic measures in PRAAT [28], in part specifically automated by scripts, which were then in part visually cross-checked for accuracy. On occasion when the PRAAT LPC algorithm failed to predict accurate formant values, such as for some quiet calls, or those with a high F0, automated measures were visually/manually assessed based on spectrograms. The following acoustic parameters were measured (details in electronic supplementary material of how measures were extracted, electronic supplementary material, figure S4). **F0:** fundamental frequency (Hz) values in the measured BUs were taken as median values across the whole BU applying the PRAAT cross-correlation algorithm, with a ceiling set at 500 Hz for grunts, and otherwise at 2000 Hz. **F0 sd**: accounts for standard variation of F0 (Hz) for the overall variance in the BU, relating to F0 slope. **F1** and **F2**: give the estimates for the first two formant centre frequencies (in Hz) in the frequency spectrum. The first and second formants are indicative of the first eigen frequency of the tube (i.e. vocal tract) describing the resonance of the air column in the tube. With respect to articulatory configurations of the vocal tract, F1 correlates inversely with vocal tract length; F2 responds most to tongue fronting [11,33,34]. **F1** and **F2** were measured using PRAAT standard Formant (burg) algorithm with five formants and 7000 Hz maximum. Resulting values were cross-checked manually/visually (by T.B.) using a combined plot of a spectrogram with 25 msec Hamming-Window and the FFT spectrum showing previously determined algorithm based values of F0, F1, F2. **COG**: centre of gravity corresponds to the spectral centroid as the weighted mean of the amplitudes in the spectrum. We applied COG to the low pass filtered (less than 8 kHz) signal. **HNR**: harmonics to noise ratio expresses the degree of acoustic periodicity and is expressed in dB. The lower the value, the more equal is the energy of both harmonics and noise, indicating a more noisy-sounding signal. **Intensity slope**: is the slope of the intensity at voice onset, i.e. abruptness of the sound onset intensity. It is measured as the amplitude difference in a fixed 10 ms window after 50 ms following the manually defined onset of the BU. The intensity was set with a minimum periodicity frequency of 200 Hz. **Duration**: of the BU in milliseconds. F0 sd, HNR and COG were measured using PRAAT standard settings.

### (e) Statistical analyses

In order to meet required assumptions of statistical procedures, appropriate variable translation and transformations (*z*-scaling and log-transformation) were conducted to obtain symmetrical distributions prior to the analysis. Also, we tested for collinearity issues between our predictor variables by computing the variance inflation factor (VIF) using the function vif from the package ‘car’ [35]. Collinearity was not an issue (VIF of all predictor variables <3).

#### (i) Principal component classification of four call types

To determine whether call types in the highly graded vocal system of chimpanzees can be clustered using an automatic classification approach, we conducted a principal components analysis (PCA) using R-function prcomp and standard rotation [36]. The correlations between covariate parameters had been determined and tested on the unaltered values in a first step. We included eight acoustic parameters to assess clusterability.

#### (ii) Discriminant classification of four call types

To determine the accuracy of classification of chimpanzee vocalizations into four broad call types: hoos, grunts, barks and screams, according to our labelling of call types, we conducted a permuted Discriminant Function Analysis permuting call types within subjects (‘pDFA’, [37]). This accounts for non-independence of the calls due to repeated recordings of the same subjects. See table 1 for the sample used.

We conducted two permuted discriminant function analyses (pDFA). pDFA1 included all eight acoustic variables assessed through acoustic analysis that described the temporal and frequency distribution characteristics of each breath unit (electronic supplementary material, table S3). pDFA2 included only articulatory variables (lip and jaw positions assigned from visual inspection of videos). As there was a smaller sample size for articulatory measures, to ensure comparability of the results of pDFA1 and 2 results, we repeated pDFA1 with a permuted, randomly selected comparable sample size (pDFA3, electronic supplementary material, table S3). To determine if acoustic and articulatory measures captured similar or different classification dimensions, we ran a fourth analysis including both acoustic and articulatory measures (pDFA4, electronic supplementary material, table S3). To balance the contribution of the individuals to the dataset used to derive the discriminant function, we included only one randomly selected call per individual and call type and also only individuals for which calls from each call type were available (pDFA1: *N* = 6 callers; pDFA2: *N* = 8 callers). To prevent the result from unduly depending on a particular random selection we created 100 such random selections and averaged the result. We based our assessment of the discriminability of the four call types on the percentage of correctly cross-classified calls and used 10 000 permutations to estimate the *p*-value for discriminability using one randomly selected call per individual per call type. All the remaining calls were then cross-classified using the derived discriminant functions. The pDFAs were conducted in R (v. 4.0.3 (10 October 2020); [38]) using the function for crossed designs (provided by R. Mundry) which is based on the function lda of the R package MASS [39].

Whereas PCA tries to maximize the variation for the individual variables (acoustic parameters), standard linear discriminant analysis (LDA) seeks to maximize the variation between the classes (call types). To determine the approximate loadings of different variables onto the discriminant functions, we conducted a LDA, in R using the MASS package [39], which was not possible using a pDFA.

## 3. Results

### (a) Principal component classification of four call types

Using a deliberately small acoustic feature set derived from human vocal analysis, the results of the PCA showed categorization of call types with the first four principal components accounting for 77% of the variance (table 2). The PCA showed that the most influential acoustic parameters loading onto the first principal component, F0, F1, F2, COG, were all related to the ‘pitch’ and spectral features of the calls.

**Table 2.** Principal component classification of four chimpanzee call types using acoustic variables, showing the principal component loadings and the proportion of variance explained by each principal component.

	PC1	PC2	PC3	PC4	PC5	PC6	PC7	PC8
centre of gravity	−0.323	0.388	−0.453	0.160	−0.391	0.236	0.534	0.139
harmonics to noise ratio	−0.106	−0.677	0.268	−0.054	0.012	0.030	0.673	−0.027
intensity slope	−0.123	−0.085	0.187	0.961	0.033	−0.074	−0.099	0.056
F0 sd	−0.307	−0.139	−0.583	0.021	0.662	−0.324	0.048	−0.025
duration	−0.235	−0.532	−0.352	−0.006	−0.245	0.519	−0.455	−0.026
F1	−0.536	0.209	0.280	−0.056	0.085	0.139	−0.028	−0.748
F2	−0.491	0.139	0.378	−0.161	0.300	0.267	−0.092	0.632
F0	−0.435	−0.127	0.048	−0.139	−0.501	−0.687	−0.184	0.127
SD	1.60	1.25	1.04	0.98	0.82	0.78	0.632	0.41
proportion of variance	0.32	0.19	0.14	0.12	0.085	0.076	0.051	0.02
cumulative proportion	0.31	0.51	0.64	0.77	0.85	0.93	0.98	1.00

**Figure 2.**
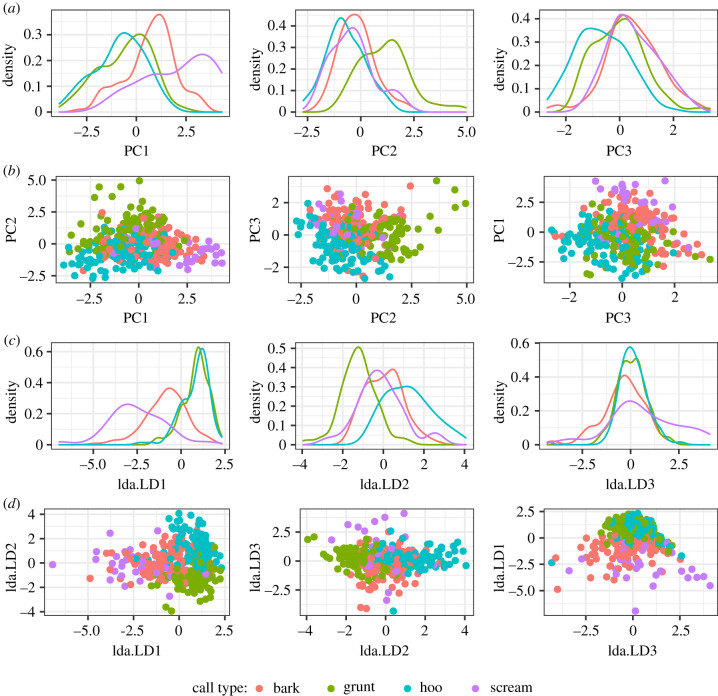
Principal component analysis clustering four chimpanzee call types. (*a*) The distribution of the maximum variation between call types across the first three principal components. (*b*) PC1 loads principally on fundamental frequency and formants, PC2 on HNR and duration, PC3 on COG and F0 sd. (*c*) The distribution of the maximum variation between call types across the first three discriminant functions in the linear discriminant analysis including acoustic variables (LDA). (*d*) LDA: discriminant function loadings for functions 1–3.

Thus, high pitched screams and low pitched hoos separated along the *x* axis. The features which loaded onto the second component were the harmonics to noise ratio (HNR) and breath unit duration, separating the rough, noisy grunts (figure 2) from the other three call types. The features which loaded onto the third component were the centre of gravity and F0 sd, while the intensity slope loaded onto the fourth component. Plotting PC1 against PC2 showed discrimination of hoos, barks and screams along PC1 and clustering of hoos from grunts along PC2 (figure 2).

### (b) Discriminant classification of four call types

Both conservative pDFA demonstrated significant differentiation of the four call types (*p* = 0.001 for each pDFA), but with a variable percentage of calls correctly cross-classified. pDFA1 included acoustic variables only, and had very similar classification rates irrespective of whether the dataset included all data (pDFA 1, *N* = 719; table 3) or was a reduced dataset (electronic supplementary material, table S3): pDFA1 correct cross-validated classification (acoustic) = 61.72%; expected cross-classification = 30.75%. pDFA2 included only the lip and jaw articulatory variables and showed a slightly higher per cent of correctly cross-classified calls (pDFA2, *N* = 394, cross-classification (articulatory) = 70.54%; expected cross-classification = 35.54%). Classification including both acoustic and articulatory measures was very similar (*N* = 394, cross-classification (acoustic and articulatory) = 72.57%; expected cross-classification = 38.43%; electronic supplementary material, table S3).

**Table 3.** Permuted discriminant analysis of four chimpanzee call types using (*a*) acoustic variables with all data and (*b*) visually defined jaw and lip articulatory variables.

	acoustic	articulatory
no. correct cross classified	428.98	256.75
no. expected correct cross classified (cc)	213.74	129.37
% correct cc	61.72	70.54
% expected correct cc	30.75	35.54
*p*-value for cc	0.001	0.001
no. randomized cases/DFA	719	398
no. cases selected to construct discriminant functions	24	34

We assessed the discriminant function loadings from a series of LDAs, using the same datasets as for the pDFAs. When including only acoustic variables, the most influential variables were centre of gravity and HNR, both loaded counter to each other on the first discriminant function. This discriminated the tonal hoos from the other three call types. HNR against F0 sd and duration loaded onto the second discriminant function. This discriminated grunts from the other three call types. F0 sd against COG loaded onto the third function, discriminating screams (figure 2; electronic supplementary material, table S4). When including only articulatory variables, jaw opening loaded counter to lip rounding on the first function. Jaw and lip protrusion loaded onto the second function, and both lip rounding and protrusion loaded onto the third function (electronic supplementary material, table S4). When including both acoustic and articulatory variables, jaw opening and COG remained dominant on the first function, whereas HNR and lip protrusion governed the second function.

### (c) Formant plot

**Figure 3.**
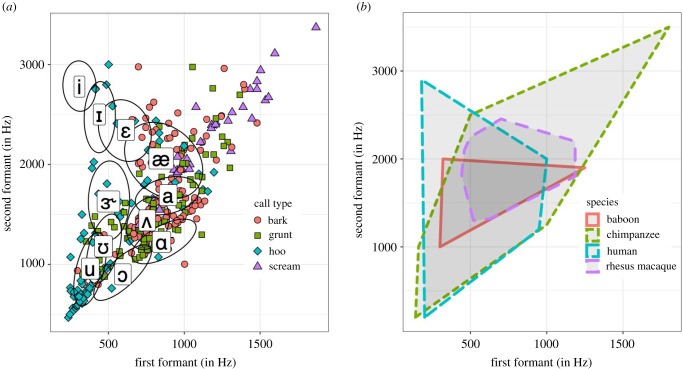
(*a*) Formant plot of current dataset (14 female/10 male chimpanzees) with superimposed human vowels (ellipses) (taken from [40]). Although there is more correlation between F1 and F2 than in humans, chimpanzee vocalizations similar to human [u], [ε] and [a] are emitted with F1 and F2 values commensurate with similar sounding human vowels. (*b*) Formant plot comparing our chimpanzee vocalizations with other primate species, with data drawn from other studies: 15 baboons [4] and one rhesus macaque [6]: interpret with caution due to expected species differences in vocal tract length and the selection of only some calls per repertoire.

We plotted F1 and F2 for each call onto a formant plot typically used in human phonetic analyses of vowels, and more recently used to assess the F1–F2 usage in monkey vocal repertoires (e.g. baboons, a macaque), following Boë *et al*. [4,7]. Figure 3 shows that the chimpanzee F1–F2 space used in vocal output overlaps substantially with that of humans. This is especially the case for low F1–low F2 ‘back vowels’ like [u, Ɔ] which are produced in humans by raising the back of the tongue and rounding the lips, as in ‘boo’ and ‘board’, respectively; likewise for ‘central’ vowels like [a, æ], which are produced in humans with a lowered tongue and an open jaw, as in ‘bark’ and ‘back,’ respectively. We found little evidence that chimpanzees are producing low F1–high F2 ‘high frontal’ vowels such as [i, I] which are produced in humans by raising the tongue tip, as in ‘bee’ and ‘bit’, respectively. Chimpanzees additionally show formant usage outside the human range in the high F1–high F2 range.

The majority of chimpanzee calls that we classed as ‘hoos’ had a low F1–low F2 and filled the human [u] vowel space. The majority of chimpanzees calls that we classed as grunts varied from low to mid F1–F2 positions, taking up human vowel spaces congruent with [a, ɑ]. Barks overlapped in the F1–F2 substantially with grunts, but tended to occupy more central F1–F2 than lower F1–F2 positions, such as [æ, a]. Screams occupied high F1–F2 positions, outside of the human range used for speech but partly overlapping with the formant range for human screams. In human screams, F1 shifts up to 800–1200 Hz and F2 shifts up to 1400–2100 Hz [41].

### (d) Comparative primate formant plot

Superimposing the non-human primate formant space with that of American English speakers [40], we find that the formant usage of chimpanzees, and of primate species investigated in previously published studies (e.g. a rhesus macaque [6], baboons and other monkeys [4]), overlaps with that of humans (figure 3). However, none of the three species' usage overlaps fully with the human formant space. Chimpanzee vocalizations encompass two of the three extremes of the formant space [u] and [a], with no non-human primate to date achieving the third extreme observed in human speech, [i].

## 4. Discussion

We could distinguish the four broad call types (hoos, grunts, screams and barks) in the chimpanzee vocal repertoire using acoustic parameters of spectral and temporal features in a PCA and pDFA. Likewise, we could distinguish the four call types when only visual articulations were included in a pDFA, indicating that the characterization of lip and jaw movements is sufficient for distinguishing hoos, grunts, screams and barks.

### (a) Principal component analysis of acoustic variables

The principal component analysis clustered the four broad chimpanzee call types, even though the chimpanzee vocal repertoire is a highly graded system [29]. Key contributing acoustic features were formant structure, fundamental frequency (F0), and noisy versus tonal characteristics. Screams have higher formants and F0 than hoos and grunts, with barks overlapping screams in the mid range (figure 2). Grunts are noisier and rougher than hoos or screams, but overlap with barks. Specifically, the weight of HNR discriminating grunts points here to a modulation of the voice source as a separate, second dimension in the acoustic call space of chimpanzees. Barks were somewhat distinguished from other call types by the centre of gravity pointing to a higher compact energy with denser harmonics than screams. Using modulation of voice quality from the voice source to facilitate discrimination of call types is typical across primates, i.e. the modulation or alteration of the voice with emphasis on characteristics beyond fundamental frequency (pitch), namely HNR (breathiness and roughness) and higher spectral energy (timbre), etc. In humans, it is important to keep in mind that voice quality contrast not only plays a role in distinguishing emotions (e.g. sadness versus disgust) or socio-pragmatic meanings (e.g. friendly polite versus cool dismissive), but can be used to gain linguistic phonetic contrasts, such as to distinguish between two different words (‘take’ versus ‘tape’) or grammatical categories (e.g. tense markers ‘take’ versus ‘took’) [42]. The language Taa (aka !Xóõ; ISO 639-3: nmn) spoken in Namibia and Botswana is a case in point that employs contrasts between a harsh, rough voice, breathy voice, creaky voice and the regular modal voice [43]. In tone languages, voice quality is often interwoven with lexical or grammatical contrasts in pitch or melody [42]. Thus, in human language and speech, voice quality differences can be discriminatory.

### (b) Discriminant function analysis of acoustic and articulatory analyses

For the permuted discriminant analyses with acoustic measures only, discriminating acoustic features included voice quality changes such as roughness and noisiness. We also found cross-classification accuracy when using only the video-coded articulatory variables of lip rounding, lip protrusion and jaw opening such that grunts, hoos, barks and screams showed significant correct classification. Surprisingly, the acoustic variables did not improve the pDFA call classification substantially beyond that achieved with only the lip and jaw articulatory variables.

This finding suggests that our nine frequency spectrum and temporal acoustic parameters, which theoretically should capture acoustic variation related to articulatory movements, such as formant shifts, did not do so as well as expected. There are several possible explanations for this result. First, the high fundamental frequency, observed particularly in screams and barks, poses problems for accurately measuring higher formants. Second, background insect and bird noise in dense tropical forest can compromise automated acoustic measurements, particularly for quieter vocalizations such as hoos and grunts. Even with high-end audio recording equipment, these problems have traditionally hampered acoustic analyses from wild animal data [435]: to date, when using automated measures, values given for each acoustic variable for each call need verification by eye, making acoustic analysis of primate calls slow and laborious [21]. We used this approach after extracting audio files from video. Using video was necessary for the purposes of this study, to compare visual articulatory measures with acoustic measures. However, audio quality may be less pronounced than calls recorded from directed microphones typically used in animal acoustic studies. In species which use lip and jaw articulations to modify vocal production, and which also process the associated visual articulartory cues [44], video-coded articulations may offer a reasonable way to improve accuracy of vocal characterization, especially when direct measurement of formants is problematic. Third, our results beg the question whether traditional primate acoustic analyses miss some important discriminatory features, particularly when formant analyses are not included, as noted by Boë *et al*. [4]. Fourth, we did not include comprehensive measures of changes to the vocalization within the breath unit. Some vocalizations, for example, change jaw opening and lip rounding within the breath unit, particularly in barks and some screams, and the sound emitted is suggestive of formant shifts across the breath unit (for example as seen in diphthongs in human speech). Hence, accuracy in fully characterizing variation within and between BU can still be improved.

### (c) Chimpanzee formant patterns

Chimpanzee articulations of jaw opening, lip protrusion and rounding result in similar changes in formant space to those observed in human vowels. This provides further evidence that the acoustic principles of human supra-laryngeal tract can be readily applied to other primates, corroborating results from Boë *et al*. [4]. Furthermore, both sexes of chimpanzees use lip and jaw articulations to create several vowel-like sounds that distinguish call types in their vocal repertoire. Mapping of the chimpanzee formant measures onto the human F1–F2 space used for vowels in American English in female speakers [40] revealed that chimpanzees cover two of the three extremes or ‘apexes’ of the human vowel space, [u,a], but do not reach the third apex [i]. Calls that we classified as ‘hoos’ occupied one apex, the extremely low F1–F2 space characteristic of human ‘back’ vowels [u]. Hoo vocalizations are emitted with a limited jaw opening, and extensive lip protrusion and rounding (figure 1). Calls that we classified as grunts and barks reached the high F1–mid F2 space characteristic of the second apex in the human vowel triangle and vowel [a]. Grunts and barks are typically emitted with little lip rounding or protrusion and variable jaw position. Screams are emitted with mid to open jaw and open or retracted lips, and produce high F1–F2 formants. Some screams extend beyond the human vowel space, likely because these have a high fundamental frequency such that F0 moves into the vicinity of F1.

As well as demonstrating that chimpanzees are not reaching [i] formant characteristics, the variation observed within the four broad call types suggests that further acoustic differentiation of call variants is possible. Indeed, chimpanzee vocal production studies examining variation within one of the four broad call types repeatedly show systematic variation of acoustic properties with context specificity, whether in the bark [29], hoo [45], scream [46] or grunt system [18]. Thus, alarm barks can be acoustically discriminated from hunt barks [29], rest hoos from alert hoos [45], and formants add to discrimination of grunts given to high or low preference foods [18]. However, only the latter study has included formant analyses. Future studies may find that context-specific variants of barks or hoos also occupy different F1–F2 space.

### (d) Comparative analysis of formant usage across primates

Comparing the formant space used by chimpanzees with that of published data from monkey species and humans [4], we see for chimpanzees the formant space of back vowels [u, Ɔ] is potentially extended compared to that of the Old World monkey species, reaching human formant range. Low F1–F2 is achieved by extending a narrowed vocal tract (e.g. [4]). Chimpanzees potentially achieve this narrowing through extended lip protrusion and rounding. Whether greater tongue mobility (figure 1) is also involved, as occurs in human vowel production, but in the absence of larynx lowering [47] is an outstanding question. As we were unable to reliably measure tongue mobility from video, it is unclear how much tongue mobility contributes to the formant patterns. However, photos and videos suggest tongue mobility in vocal and non-vocal contexts is non-negligible, for example showing concavity during screaming and retraction during yawning (electronic supplementary material, video S1 and figure S5). Boë *et al*. [4] demonstrate that most monkeys do not use the [u] back vowel space during vocalizing (figure 3), with the exception of baboons, which may be attributed to the long baboon snout. Hence, different primate species may have expanded their vowel space in different ways. Whether this variation is through selection pressures to increase sound diversity, or through indirect causes, for example, changes to articulators occurring due to other selection pressures, remains to be examined. Nonetheless, it is apparent that divergent anatomies have created various solutions to vowel space expansion. Important to note, primates seem capable of perceiving variation in the formant patterns within a call type (baboon grunts: [16]; rhesus macaque coos: [48]), to discriminate, for instance, between kin and non-kin individuals or even between different individuals, as demonstrated in playback experiments (rhesus macaque: [49]; cotton-top tamarin: [50]; chimpanzees: [51]).

To date, comparative analyses suggest that humans use three vowel space extremes: [i], [u] and [a]. Chimpanzees reach two of these extremes, [u] and [a], whereas to date monkey species have been reported to reach a maximum of one of these extremes, either [u] (baboons: [7]) or [a] (Diana monkeys: [52]), but no non-human primate species has been shown to reach [i] [4,6]. In sum, formant space overlaps across primate species; however, extending the formant space to include the third extreme [i] used in human speech might be a derived capacity in the hominid lineage. Further primate studies are required to confirm this idea. Within the limitation that most primate studies have conducted formant analyses on only small parts of each species' vocal repertoire rather than offering a whole vocal repertoire formant analysis, it is currently not possible to rule out whether some monkey species reach more than one of the three human vowel extremities. Thus, quantitative whole vocal repertoire monkey studies are required to confirm whether a vocal repertoire that encompasses two of the human formant extremes is a derived trait in hominoids. It might also be that other primates use formant space not used in human speech, as indicated in figure 3.

Boë *et al.* [7] pointed out that human speech may show less F1–F2 correlation than baboon vocalizations. Given the slightly expanded vowel space in chimpanzee vocalizations compared to monkeys (figure 3), chimpanzees arguably show greater release from F1–F2 correlation than baboons but not to the degree that humans have reached. This finding is suggestive of continued expansion of the vowel space that overlaps with human vowel space through the primate lineage, although assessment in more primate species would be required to confirm this hypothesis.

## 5. Conclusion

Chimpanzee vocalizations can be broadly classified into four major call types: hoos, grunts, barks and screams, using either discriminant or principal component classification approaches. Classification was possible even though the chimpanzee vocal repertoire is a graded system [29]. When using standard acoustic measures, our analyses demonstrate that call type discrimination can be achieved through the use of two acoustic dimensions, variously using voice source and supra-laryngeal filter modification. We also classified the four call types using novel articulatory parameters that visually characterize the lip and jaw movements, which shape the spectral and temporal features of each call. Thus including articulatory parameters might be a useful additional classification approach for species that live in particularly noisy environments.

Our comparative analyses suggest differential vowel space usage across primate species. All primate species examined to date demonstrate overlap in vowel space usage with humans, particularly in the space equivalent to the human central space, representing [a] and schwa vowels. There is also indication that non-human primates may use vowel space *not* used by humans, particularly when both F1 and F2 are high.

Our comparative results including data from previous studies suggest that chimpanzees share both a larger and more overlapping vowel space with humans than monkeys species. Presumably the capacity to generate a larger vowel space creates the potential to create more divergent vocal signals and hence more diverse vocal messages. To determine whether, and if so how, supra-laryngeal articulatory capacities and hence vowel space usage change with vocal complexity and the evolution of language, however, will require broad examination of vowel space usage across the vocal repertoire in more primate species. One part of the vowel space not yet demonstrated to occur outside of humans is the space usage that requires high frontal tongue positioning, as in [i]. Whether this tongue movement in a vocalization remains outside the articulatory capacities of non-human primates requires further examination.

The difficulties of directly comparing repertoire sizes across species are well documented, being subject to problems such as whether researchers are ‘lumpers’ or ‘splitters’. The former condense graded call types into fewer, broader call types, as here, while the latter expand repertoires into more, narrower call types. Examining formant space and its usage across species' repertoires may facilitate cross-species comparison, giving a standardized and quantitative metric for comparison that also encompasses the human vocal repertoire. What socio-ecological factors may have selected for vowel space variation through the primate, and more specifically, the hominoid lineage is a separate and fascinating research question.

## Data accessibility

The R script, PRAAT script and data files are included in the electronic supplementary material [53].
