# Correction: ‘Chimpanzee vowel-like sounds and voice quality suggest formant space expansion through the hominoid lineage’ (2021), by Grawunder *et al.*

**DOI:** 10.1098/rstb.2023.0319

**Published:** 2023-11-20

**Authors:** Sven Grawunder, Natalie Uomini, Liran Samuni, Tatiana Bortolato, Cédric Girard-Buttoz, Roman M. Wittig, Catherine Crockford

**Affiliations:** ^1^ Department of Human Behavioural Ecology, Max Planck Institute for Evolutionary Anthropology, Deutscher Platz 6, 04103 Leipzig, Germany; ^2^ Department of Linguistic and Cultural Evolution, Max Planck Institute for Evolutionary Anthropology, Deutscher Platz 6, 04103 Leipzig, Germany; ^3^ Department of Empirical Linguistics, Goethe University, 60323 Frankfurt am Main, Germany; ^4^ The Ape Social Mind Lab, Institut des Sciences Cognitives, CNRS, 67 Boulevard Pinel, 69675 Bron, Lyon, France; ^5^ Department of Human Evolutionary Biology, Harvard University, Cambridge, MA, MCZ 540B, USA; ^6^ Tai Chimpanzee Project, Centre Swiss des Recherches Scientifiques, 01 BP 1303, Abidjan, Côte d'Ivoire

**Keywords:** evolution of language, formants, primate, speech, chimpanzees, hominoid

*Phil. Trans. R. Soc. B* **377**, 20200455 (Published online 15 November 2021). (https://doi.org/10.1098/rstb.2020.0455)

Following discussion about formant measures, we amend the following aspects of the original paper. Note that the key results remain unchanged. For the first result, broad chimpanzee call types, hoos, grunts, screams and barks, though graded, can be discriminated using acoustic parameters or only lip and jaw articulatory measures. For the second result, the chimpanzee formant space appears expanded compared to African and Asian monkeys, embracing two of the three F1/F2 formant space extremities used in humans [1]. Specifically, chimpanzee compared to monkey vocalizations encompass both [u] and [a] extremities, rather than only one or no extremities. However, the third extremity [i], appears outside the capacity of the few primate species examined to date, and may represent a derived capacity in the hominin lineage.

## 1. Chimpanzee formant measures

In Grawunder *et al.* [12], we took the position recently emphasized by Herbst & Story [2], to distinguish formants as properties of an acoustic frequency spectrum [3] from vocal tract resonances (R1, R2, R3,…) that are indicated by formants.

In general, measuring or estimating formant centre frequencies is error prone, especially in calls with higher fundamental frequencies owing to a wider harmonic spacing and a lack of non-periodic components [4]. When F0 is unmodulated, such as for some calls in this dataset, measuring F1 can be problematic [4]. Whilst in the original paper we acknowledged this issue and opted to treat the full dataset uniformly, here we demonstrate a different approach, and instigated three correctional measures (figure 1*a*). First, as formant measurements are particularly problematic for calls with a high F0 and widely spaced harmonics, we remove calls with F0 > 800 Hz (31 screams, 29 barks, one hoo, one grunt). Whilst this removes most screams (which typically have a high F0), the formant distribution of other call types across the formant space remains largely unchanged. The result is that most of the data points now fall within the formant space for human speech, defined by Boë *et al*. [1] as the maximum acoustic space (MAS triangle). Second, remaining cases where F0 ≈ F1 were also removed. Both correction steps result in a reduced formant dataset of 302 of 424 calls. Third, according to common practise in the field [8], we used a formant-guided approach of [u]-like calls, narrowing the region of interest such that F1 < 600 Hz and F2 < 1000 Hz. Note, while most call units labelled as ‘hoo’ sounds show consistent formants, others show a wider range across call type areas. Closer inspection suggests that in call bouts traditionally defined as being repeated units of the same call type, such as in the ‘build-up’ phase of pant hoot vocalizations which is constructed of panted hoos (e.g. [9]), the quality of the hoos can change to a more open-mouthed articulation as the bout progresses. Future studies should consider more fine-grained analyses of repeated units within call bouts.

**Figure 1.**
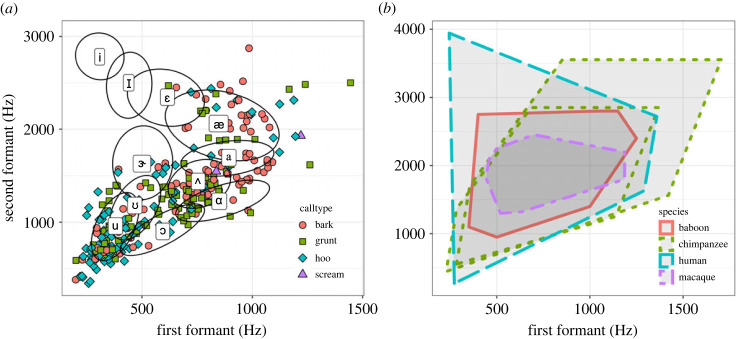
Chimpanzee formant space use: (*a*) revised plot showing chimpanzee vocalizations according to F1 and F2 values. Formants follow three constraints. Calls were included when: (i) F0 < 800 Hz; (ii) for [u]-like vocalizations, F1 < 600 Hz and F2 < 1000 Hz; and (iii) F0 ≠ F1. (*b*) Revised plot comparing normalized formant measures across four primate species, including humans, using macaque vocal tract length (VTL) = 11.4 cm as reference [1]. Human VTL is taken as that for women at 15.5 cm [5]. Chimpanzee VTL is taken as 16.2 cm (larger dotted green shape) [6] and 13 cm (smaller dotted green shape) [7]. The normalized formant values for macaques and baboons is taken from Boë *et al*. [1]. See the electronic supplementary material for additional sound files and data. (Online version in colour.)

## 2. Normalization of formants across primates

Given that primates have different vocal tract lengths (VTL), which affect formants, we followed Boë *et al.* [1] by correcting for species' VTL. We normalized formant value F by the relation of the two VTLs l1 and l2, so that Fl2 = (l1/l2) × Fl1, using the rhesus macaque VTL = 11.4 cm as the reference (l2) [1]. In the original paper, we did not normalize the data as for most primate species there are no or few actual VTL measures, including for chimpanzees. Most are estimated, including in Boë *et al*. [1].

Here, we normalize chimpanzee VTLs using two VTL measures for our sample of adult chimpanzees: (i) of 16.2 cm, following Nishimura [6], and (ii) 13 cm, following Lieberman [7] (figure 1). Chimpanzee VTL measures are extremely rare in general, and non-existent for live chimpanzees given that invasive measures are needed to calculate them. Hence, for (i) we took the average VTL of the three preserved chimpanzee cadavers classified as ‘adult’ in Nishimura [6], and scanned by means of magnetic resonance imaging. For humans, we use a VTL of 15.5 cm for women [8], and took the normalized formant values for macaques and baboons from Boë *et al*. [1].

Please also note that there is yet no general agreement about appropriate VTL measurement in humans. Such measures do not only vary with body size and phenotype (race) but also with posture (laying, standing) and vocal activity (resting, speaking, singing/calling) during measurement (cf. [3,8,10,11]).

## 3. Conclusion regarding chimpanzee F1/F2 space

The main conclusions of the comparative plot are unchanged. In summary, the macaque vocalizations use the central formant space. Chimpanzees to a greater extent, and baboons to a lesser extent, use vocalizations with lower F1/F2 (bottom left, [u] as in ‘boot’) and higher F1/F2 (top right, [a] as in ‘car’) than the macaque. Hence, they fill more of the formant space used in human speech. Given that chimpanzees use more of this space than the monkey species, we conclude that chimpanzees use an expanded formant space, close to that of humans. However, none of the non-human primates used the third extremity, [i] as in ‘peek’. Extending the formant space to include the third extremity, [i], used in human speech might be a derived capacity in the hominin lineage.

## 4. Principal component analysis and discriminant function analysis without formant measures

In the original paper, we demonstrated classifiability of the four main, but graded, chimpanzee call types (hoos, grunts, screams, barks). In order not to change or decrease the original dataset for the principal component analysis (PCA) and discriminant function analysis (DFA), we reran the PCA and DFA without the formant values, with the full dataset. Note that excluding calls with high F0 excludes most screams from the sample, which would limit the purpose of the PCA and DFA to determine classifiability of the four call types. Reanalysis demonstrates that classifiability of the acoustic variables remains high without F1 and F2 included (tables 1 and 2). Classifiability also remains high when only using articulatory variables of lip and jaw positions.

**Table 1.** Principal component classification of four chimpanzee call types using acoustic variables (excluding F1 and F2), showing the principal component loadings and the proportion of variance explained by each principal component.

	PC1	PC2	PC3	PC4	PC5	PC6
cog	−0.3476	0.5985	−0.0358	0.3555	−0.1816	0.6003
HNRmd	−0.2212	−0.6987	−0.0135	0.0170	0.2487	0.6329
intslope	−0.1544	−0.0305	−0.9806	−0.0609	−0.0366	−0.0925
F0sd	−0.4840	0.2092	0.1133	−0.8341	0.1074	0.0444
duration	−0.4951	−0.3293	0.1320	0.1136	−0.7462	−0.2435
F0	−0.5719	0.0227	0.0816	0.4013	0.5792	−0.4114
standard deviation	1.3962	1.2210	0.9918	0.8117	0.7508	0.5945
proportion of variance	0.3249	0.2485	0.1639	0.1098	0.0939	0.0589
cumul. proportion	0.3249	0.5734	0.7373	0.8471	0.9410	1.0000

**Table 2.** Permuted discriminant analysis of four chimpanzee call types using (*a*) acoustic variables with all data (excluding F1 and F2), (*b*) visually defined jaw and lip articulatory variables, and (*c*) both acoustic and articulatory variables.

	acoustic	articulators	articulators + acoustic
no. correct cross classified	515.42	244.84	264.86
expected correct cross classified (cc)	233.28	128.89	134.24
% correct cc	66.16	67.26	74.19
% expected correct cc	29.95	35.41	37.60
*p*-value for cc	0.001	0.001	0.001
no. randomized cases/DFA	807	398	391
no. cases selected to construct discriminant functions	28	34	34

## Data Availability

Additional sound files and data are now provided in the electronic supplementary material [12]. These supplement the files provided with the original publication [13].

## References

[1] Boë L-J, Sawallis TR, Fagot J, Badin P, Barbier G, Captier G, Ménard L, Heim J-L, Schwartz J-L. 2019 Which way to the dawn of speech? Reanalyzing half a century of debates and data in light of speech science. Sci. Adv. **5**, eaaw3916. (10.1126/sciadv.aaw3916) 32076631 PMC7000245

[2] Herbst CT, Story BH. 2022 Computer simulation of vocal tract resonance tuning strategies with respect to fundamental frequency and voice source spectral slope in singing. J. Acoust. Soc. Am. **152**, 3548-3561. (10.1121/10.0014421) 36586864

[3] Fant G. 1966 A note on vocal tract size factors and non-uniform F-pattern scalings. STL-QPSR **4**, 22-30.

[4] Alku P, Pohjalainen J, Vainio M, Laukkanen AM, Story BH. 2013 Formant frequency estimation of high-pitched vowels using weighted linear prediction. J. Acoust. Soc. Am. **134**, 1295-1313. (10.1121/1.4812756) 23927127

[5] Xue SA, Hao JG. 2006 Normative standards for vocal tract dimensions by race as measured by acoustic pharyngometry. J. Voice **20**, 391-400. (10.1016/j.jvoice.2005.05.001) 16243483

[6] Nishimura T. 2005 Developmental changes in the shape of the supralaryngeal vocal tract in chimpanzees. Am. J. Phys. Anthr. **126**, 193-204. (10.1002/ajpa.20112) 15386289

[7] Lieberman P. 1968 Primate vocalizations and human linguistic ability. J. Acoust. Soc. Am. **44**, 1574-1584. (10.1121/1.1911299) 5702032

[8] Ladefoged P. 2003 Phonetic data analysis: an introduction to fieldwork and instrumental techniques. New York, NY: Wiley-Blackwell.

[9] Fedurek P, Machanda ZP, Schel AM, Slocombe KE. 2013 Pant hoot chorusing and social bonds in male chimpanzees. Anim. Behav. **86**, 189-196. (10.1016/j.anbehav.2013.05.010)

[10] Fitch WT, Giedd J. 1999 Morphology and development of the human vocal tract: a study using magnetic resonance imaging. J. Acoust. Soc. Am. **106**, 1511-1522. (10.1121/1.427148) 10489707

[11] Dmitriev L, Kiselev A. 1979 Relationship between the formant structure of different types of singing voices and the dimensions of supraglottic cavities. Folia Phoniatr. Logop **31**, 238-241. (10.1159/000264170) 544402

[12] Grawunder S, Uomini N, Samuni L, Bortolato T, Girard-Buttoz C, Wittig RM, Crockford C. 2023 Correction to: ‘Chimpanzee vowel-like sounds and voice quality suggest formant space expansion through the hominoid lineage’. *Figshare*. (10.6084/m9.figshare.c.6823016) PMC8591386 34775819

[13] Grawunder S, Uomini N, Samuni L, Bortolato T, Girard-Buttoz C, Wittig RM, Crockford C. 2022 Chimpanzee vowel-like sounds and voice quality suggest formant space expansion through the hominoid lineage. *Phil. Trans. R. Soc. B* **377**(1841), 20200455. (10.6084/m9.figshare.c.5662240) PMC8591386 34775819

